# Draft Genome Sequence of “*Candidatus* Liberibacter asiaticus” from *Diaphorina citri* in Guangdong, China

**DOI:** 10.1128/genomeA.01316-15

**Published:** 2015-11-05

**Authors:** F. Wu, Z. Zheng, X. Deng, Y. Cen, G. Liang, J. Chen

**Affiliations:** a Department of Plant Pathology, Laboratory of Insect Ecology, South China Agricultural University, Guangzhou, Guangdong, China; b San Joaquín Valley Agricultural Sciences Center, USDA-ARS, Parlier, California, USA

## Abstract

The draft genome sequence of “*Candidatus* Liberibacter asiaticus” strain YCPsy from an Asian citrus psyllid (*Diaphorina citri*) in Guangdong, China, is reported here. The YCPsy strain has a genome size of 1,233,647 bp, 36.5% G+C content, 1,171 open reading frames (ORFs), and 53 RNAs.

## GENOME ANNOUNCEMENT

“*Candidatus* Liberibacter asiaticus,” an unculturable alphaproteobacterium, inhabits both Asian citrus psyllids (*Diaphorina citri* Kuwayama) and citrus plants. Infection of the bacterium is associated with citrus huanglongbing (HLB) (yellow shoot disease), a destructive disease in citrus production. *D. citri* transmits “*Ca.* Liberibacter asiaticus” (1). HLB was observed in Guangdong, China, >100 years ago (2). Transmission of the HLB pathogen by *D. citri* was reported in 1977 (3) before the detection of “*Ca.* Liberibacter asiaticus” in 1996 (4, 5). Because of the lack of *in vitro* culture, research on “*Ca.* Liberibacter asiaticus” has been challenging. Thanks to the development of next-generation sequencing technology, genomes of “*Ca.* Liberibacter asiaticus” strains can be sequenced directly from psyllid or plant hosts for biological study. Zheng et al. (6) sequenced the genome of “*Ca.* Liberibacter asiaticus” strain A4 from periwinkle in Guangdong. Here, we report a draft genome sequence of “*Ca.* Liberibacter asiaticus” from *D. citri* in the same geographical location.

A mandarin citrus tree (*Citrus reticulata* cv. Shatangju) infected with “*Ca.* Liberibacter asiaticus” was maintained in a growth chamber (RXZ-380A; Jiangnan Instrument, Inc., Ningbo, China) with the settings of 28 ± 1°C, 60% ± 5% rH, and 14:10 h light/dark (L:D) at South China Agricultural University in Guangzhou, China. The original source of “*Ca.* Liberibacter asiaticus” was from an HLB Shatangju tree in Boluo City of Guangdong (23°26′07″N, 114°29′56″E). Psyllids were fed on the infected citrus tree for 2 months. An individual psyllid adult was collected for DNA extraction using the DNeasy blood and tissue kit (Qiagen, Shanghai, China). Infection of “*Ca.* Liberibacter asiaticus” was monitored by the PCR method of Li et al. (7). DNA from a single psyllid sample (threshold cycle [*C_T_*], 18.1) was amplified using illustra GenomiPhi version 2 DNA amplification kits (GE Healthcare, Inc., Waukesha, WI, USA). The amplified DNA was sequenced using an Illumina MiSeq format (Illumina, Inc., San Diego, CA).

A total of 3.98 × 10^7^ reads with a mean of 251 bp per read were generated from the psyllid DNA sample. Using the whole genomes of “*Ca.* Liberibacter asiaticus” strains psy62 (8) and A4 (6) as references, a total of 5,854,876 and 5,886,489 reads, respectively, were identified using the standalone BLAST software (version 2.2.30; e-value, <10^-20^) (9). The “*Ca.* Liberibacter asiaticus” reads were collected using a Perl script. The combination of *de novo* assembly using Velvet (version 1.2.10) (10) and reference assembly using Bowtie2 (version 2.2.6) (11) generated 9 contigs ranging from 1,587 bp to 755,458 bp, with an average coverage of 1,120×. The draft genome of “*Ca.* Liberibacter asiaticus” strain YCPsy comprises 1,233,647 bp, with a G+C content of 36.5%. Annotation was performed using the RAST server (http://rast.nmpdr.org/) (12), and the YCPsy genome was predicted to have 1,171 open reading frames (ORFs) and 53 RNAs.

### Nucleotide sequence accession numbers.

This whole-genome shotgun project has been deposited at DDBJ/EMBL/GenBank under the accession no. LIIM00000000. The version described in this manuscript is the first version LIIM01000000.
